# The Tuscany Regional Network for rare diseases: from European Reference Networks’ experience to registry based organisation and management model for rare diseases

**DOI:** 10.1186/s13023-023-02947-4

**Published:** 2023-10-13

**Authors:** Federica Pieroni, Sonia Marrucci, Linda Di Pietro, Cecilia Berni, Cristina Scaletti

**Affiliations:** 1 Fondazione Toscana Gabriele Monasterio, Pisa, Italy; 2 Department of Experimental and Clinical Medicine, University of Florence, Florence, Italy; 3 Tuscany Region’s Rare Disease Network, Tuscany Region, Florence, Italy; 4 Rare Diseases, Congenital Anomalies, Birth Path and Paediatrics Network - Healthcare, Welfare and Social Cohesion Directorate, Tuscany Region, Florence, Italy; 5 Careggi University Hospital, Florence, Italy

**Keywords:** Rare diseases, Registry, European Reference Networks, Regional network

## Abstract

**Background:**

In the European Union, a disease is defined as rare when it affects fewer than 1 in 2000 people. Currently, there are up to 8000 described rare diseases (RDs), collectively affecting 30 million people in the European Union. In 2004 Tuscany region (Italy) established a Regional Network of hospital units to ensure highly specialised medical care in the field of RDs. Shortly after the Rare Diseases Registry of Tuscany (Registro Toscano Malattie Rare—RTMR) was implemented. Here we describe the analysis performed on RTMR data which has recently allowed to remap the Network based on European Reference Networks’ model.

**Results:**

Data analysis was performed on 60,367 cases registered in RTMR, regarding 628 RDs. Two-hundred and fifteen active presidia have been evaluated. The assignment of each RD to the suitable European Reference Network has been made considering not only the number of registered cases, certifications and treatment plans for each Regional Presidium but also the competence in multidisciplinary management of the patient, from diagnosis to treatment. This evaluation has led to the establishment of twenty-one Regional Coordination Centres. They aggregate and coordinate Hospital Units which diagnose and treat one or a group of related RDs. In case of wide groups of RDs, Clinical Subnets are instituted. Updated statistics regarding RDs in Tuscany, list of RDs and Coordination Centres, as well as information about single Presidia are published and freely available on a designated webpage. Regional Decrees are regularly updated according to the network evolution.

**Conclusions:**

The Rare Diseases Regional Network in Tuscany, based on the ERN model, has played a pivotal role in enhancing RD management and research. The remapping has led to a dynamic system, following not only scientific research but also the development of Presidia’s expertise. By pooling resources and expertise, the network has improved the availability and accessibility of specialized care for patients with RDs. Collaborative efforts, data sharing, and standardized registries are crucial for advancing RD research, improving diagnosis and treatment, and ultimately enhancing the quality of life for individuals living with RDs.

## Background

A disease is defined as rare when it affects fewer than 1 in 2000 people in the European Union, according to European definition [1]. Currently, there are up to 8000 described rare diseases (RDs), collectively affecting 300 million people worldwide, 30 million in the EU [2]. Therefore, although individually rare, it is estimated that 6–8% of the European population suffers from a RD [3].

RDs are often misdiagnosed, or the diagnosis is delayed, due to the heterogeneity of their clinical signs and symptoms [4–6]. Moreover, these conditions are often severe, multi-systemic and have a significant impact on life expectancy [7, 8]. Patients affected by RDs need a highly specialised, multidisciplinary and personalised medical care both to ensure a correct diagnosis and access to proper treatments. Consequently, RDs represent a major public health issue [9, 10] but their real burden is difficult to estimate: they are often differently defined according to specific jurisdiction [11] and there is a paucity of reliable population data [12, 13].

From this perspective, a public healthcare system should develop a network of Centres of particular experience in the multidisciplinary management of patients affected by RDs. The network should be monitored and updated continuously to guarantee the collection and sharing of epidemiological, clinical and therapeutic data.

In accordance with Italian Ministerial Decree 279/2001 [14], in 2004 Tuscany, an Italian region of 3 649 447 inhabitants (source: Italian National Institute of Statistics as of 28th February 2023) established a new model of Regional Network for RDs, based on hospital units (Presidia) with previous expertise in the diagnosis and management of RDs. This network was built on Regional Coordination Centres which had to identify, supervise and optimise the multidisciplinary care for each RDs or group of RDs, supporting the cooperation among Presidia.

In 2005 the Registro Toscano Malattie Rare (Rare Diseases Registry of Tuscany—RTMR) was implemented. This tool became essential to effectively collect the clinical records regarding patients affected by RDs in Tuscany. The analysis of those data has allowed to improve therapeutic and diagnostic pathways (PDTA), supporting the health planning in the field of RDs. Moreover, it has been a valuable backing to promote scientific research.

On a European level, the RDs network is based on 24 European Reference Networks (ERN). They were launched in 2017, involving highly specialised healthcare units from over 300 hospitals in 26 Member States. As of January 2022, the total number of ERN members had reached almost 1500.

In Tuscany, the Regional Committee resolution 133/2020 aimed to structure the Rare Diseases Regional Network analogously to the European Reference Networks (ERN).

Currently, each Presidia involved in the Network can update the RTMR according to their expertise and their consequent position in the Network: a hospital unit can be qualified to insert in the Registry a diagnosis and/or certification of RD, to issue a treatment plan and/or to perform follow-up visits.

The designated website [15] displays the list of Presidia specialised in the care of each RD, their role in the Regional Network and an updated clinical records listing for each Unit.

Here we describe the analysis performed on the RTMR data which has led to the remapping of the Regional Network of Rare Diseases in Tuscany on the basis of ERNs model. The revised network has been conceived as a dynamic system, subject to frequent updates, keeping up with the evolution of scientific research and innovation in diagnosis and management of Rare Diseases.

## Methods

The study, launched in 2020, was carried out by the Registro Toscano Malattie Rare (RTMR), managed by Fondazione Toscana Gabriele Monasterio (FTGM) for medical and public health research in Pisa, in collaboration with Tuscany Region. The data analysis aims to identify the Regional Coordination Centres for Rare Diseases and Clinical Subnets, as well as the coordinators of these Centres, remapping the Network on the basis of ERN networks’ structure. Through RTMR, the detection and analysis of cases is ensured, supporting health planning and management. RTMR back-office functionalities have been developed to support the operational workflow and functional use of data.

In RTMR the following information are coded and maintained:

- RDs included in 2017 Ministerial Decree [16] (DPCM) and non-exempt diseases (EXTRA DPCM); the latter are recorded in the Registry for epidemiological purpose.
- Tuscany Hospital Units (Presidia) with expertise in at least one RD diagnosis and/or therapy and/or follow-up.
- Hospital units identified as Coordination Centres and Clinical Subnets Centres by Tuscany region.
- Users (physicians belonging to a Presidium) and special users (Coordination Centres managers and Clinical Subnets Centres managers).
- RTMR roles label every Unit involved in the network and specify the level of expertise on a single RD, in order to establish a continuously growing network of accredited centres. Provided RTMR roles are: DIAGNOSIS, which is necessary to register a diagnosis in RTMR; CERTIFICATION, to confirm diagnosis in order to obtain exemption from medical expenses; THERAPY, a role assigned to centres that are allowed to insert a treatment plan and to release it to patients.
- Records of patients who have received a RD diagnosis since year 2000 (CASES).
- Digital certifications related to a diagnosis (CERT), introduced in May 2017.
- Digital treatment plans related to a diagnosis (TP), introduced in May 2017.

Each RTMR CASE can be registered in two different ways:

- First diagnosis (FD).
- Follow-up visit (CONT): the medical visit confirming a FD made by another Presidium or a follow-up visit.

### Dataset

The dataset is built extracting all CASES recorded by each Presidium from 2014 to May 2020 and related CERT and TP released in the timeframe between June 2017 and May 2020. This timeframe was chosen to have an overview at the moment of the start of the network reorganisation process. The structure of the initial dataset is shown in Tables 1 and 2. As shown, the first columns list the RDs, identified by exemption code and group. The three following columns display the National proposal for RD attribution to ERNs and the ORPHAcode specified in the subsequent Ministerial Decree, while a subsequent column reports the identified ERN on a European level.

**Table 1:** Dataset, rare diseases’ classification

National RD coding			National (ISS) proposal for RD attribution to ERN's				Data from European file (specificcriteria_en.xls)			
Exemption code	RD name	Group	ERN 1st national candidate	ERN 2nd national candidate	ERN 3rd national candidate	ORPHA codes national	Identified ERN	Main thematic group	Sub-thematic areas	Rare or complex disease(s), condition(s) or highly specialised intervention(s)

*RD* rare disease, *ERN* European reference network

**Table 2:** Dataset, data registered in RTMR

Single presidium RTMR data							All presidia RTMR data			Single presidum (%)			
Presidium (Healthcare Company—Department—Unit)	Tot CASES from 2000 (FD OR CONT)	Residents	Tot CASES from 2014 (FD OR CONT)	Residents	Tot CERT from 27/5/17	Tot TP from 27/5/17	Tot CASES	Tot CERT	Tot TP	% CASES	% CERT	% TP	Calculation notes

*RTMR* = Registro Toscano Malattie Rare (Rare Diseases Registry of Tuscany), *FD* first diagnosis, *CONT* follow-up visit, *CERT* certification, *TP* treatment plan

### Presidia evaluation

The evaluation and verification of ERNs are based on the proposals of both the Italian Regions and the Italian National Institute of Health, but also taking into account the ORPHAcode. Each rare disease is identified within a definite file available in ERNs’ documentation, named “specificcriteria_en” [17], through the ORPHAcode. Nevertheless, the RD may appear in the file with several other captions, or it may be allocated in different ERNs. In these cases, the choice of the specific ERN, and therefore the identification of the correct Coordination Centre, is made considering requirements such as Main Thematic Group, Sub-thematic Areas and Rare or Complex Disease(s), Condition(s) or Highly Specialized Intervention(s). Other criteria are considered: the total number of rare diseases’ Diagnosis, the total number of Certifications and Treatment Plans issued by Presidia through the Registry in the chosen timeframe and the Presidia’s previous involvement in ERN.

In Table 2 Presidia activity on each RD is monitored, calculating each Presidium share (%) of CASES/CERT/TP inserted in RTMR (grey fields). The percentages of Cases (CASES), Certifications (CERT) and Treatment Plans (TP) for each RD and for each Presidium were then calculated as follows:

- %CASES = nCASESpresidium/nCASEStot
- %CERT = nCERTpresidium/nCERTtot
- %TP = nTPpresidium/nTPtot

Thus, it possible to highlight the Presidium that meet the following requirements:

- BEST CASES = Presidium with the highest number of cases
- TOP CASES = Presidium with number of cases ≥ 5% of total

A 5% threshold is established after data simulation: this value allows to include Presidia with fewer but statistically significant cases. For each RD, the BEST CASES, BEST CERT and BEST TP labels are assigned to one or more Presidia. In the same way, TOP CASES, TOP CERT and TOP TP labels are assigned. Data are shared with the Tuscany Region for an additional evaluation. Data are then summarised in twenty-four Pivot Tables, one for each ERN. The rows are organised by RDs possibly assigned to said ERN, columns by Presidia and data fields display TOP and BEST labels for each category (cases, certifications and treatment plans). The definitive ERN for each RD is chosen considering the Hospital Unit with BEST labels for cases, certification and treatment plans but also taking into account disease pathogenesis and clinical features, such as most frequent organ involvement.

### Simple case example: Kallmann syndrome

Kallmann syndrome (KS) is a genetic disorder characterized by congenital hypogonadotropic hypogonadism (CHH) due to gonadotropin-releasing hormone (GnRH) deficiency, associated with anosmia or hyposmia due to hypoplasia or aplasia of the olfactory bulbs [18]. In this case, both the Italian National Institute of Health evaluation and the data from the European file (ORPHAcode, thematic group, spectrum of clinical expression) suggest Endo-ERN as the most suitable network for Kallman syndrome at a European and National level (Table 3).

**Table 3:** Kallman syndrome example

National RD coding			National (ISS) proposal for RD attribution to ERN's				Data from EUROPEAN file (specificcriteria_en.xls)			
Exemption code	RD NAME	Group	ERN 1st national candidate	National candidate	National	Proposal	Identified ERN	Main thematic group	Sub-thematic areas	Rare or complex disease(s), condition(s) or highly specialised intervention(s)
RC0020	Kallmann, sindrome di	Malattie delle ghiandole endocrine	ENDO			478	ENDO	Sexual development and maturation		Kallmann syndrome

*RD* rare disease, *ISS* = Istituto Superiore di Sanita (Italian National Institute of Health), *ERN* European reference network

Consistently, Table 4 shows how, in Tuscany, Presidia with the highest rates of cases, certification and treatment plans are Endocrinology Hospital Units, verifying and confirming those recommandations for the Tuscany Region.

**Table 4:** Kallman syndrome: data from Presidia in Tuscany

Single presidium RTMR data								All presidia RTMR data			Single presidium %		
Presidium (Healthcare Company-Department/Unit)		Tot CASES FROM 2000 (FD OR CONT)	Residents	Tot CASES from 2014 (FD OR CONT)	Residents (%)	Tot CERT FROM 27/5/17	Tot TP from 27/5/17	Tot CASES	Tot CERT	TOT TP	% CASES	% CERT	% TP
Presidium 1	Endocrinology and andrology	29	26	18	94	3	1	62	16	17	29.0	18.8	5.9
Presidium 1	Genetics	2	2	1	100	0	0	62	16	17	1.6	0.0	0.0
Presidium 1	Endocrinology	8	7	2	50	1	1	62	16	17	3.2	6.3	5.9
Presidium 1	Pediatric gynecology	3	2	1	100	0	0	62	16	17	1.6	0.0	0.0
Presidium 2	Endocrinology 1	7	2	3	33	0	0	62	16	17	4.8	0.0	0.0
Presidium 2	Endocrinology 2	11	6	11	55	8	8	62	16	17	17.7	50.0	47.1
Presidium 2	Molecular genetics	9	2	9	22	0	0	62	16	17	14.5	0.0	0.0
Presidium 2	Clinical genetics	1	1	1	100	0	0	62	16	17	1.6	0.0	0.0
Presidium 2	Pediatric endocrinology	4	3	3	100	3	7	62	16	17	4.8	18.8	41.2
Presidium 3	Congenital bone diseases	1	1	1	100	1	0	62	16	17	1.6	6.3	0.0
Presidium 3	Genetics	8	6	2	100	0	0	62	16	17	32	0.0	0.0
Presidium 4	Genetics	1	1	1	100	0	0	62	16	17	1.6	0.0	0.0

*FD* first diagnosis, *CONT* follow-up visit, *TP* treatment plans, *CERT* = certifications

In the table only most relevant Presidia for Kallman syndrome are shown. Inactive Presidia at the moment of data analysis have been omitted

### Complex case example: polymyositis

Polymyositis is a rare immune-mediated inflammatory myopathy characterized by symmetric proximal muscle weakness, elevated muscle enzymes and myopathic findings on electromyography [19]. Given the non-specific clinical features, the disease should be distinguished from similar rare entities such as dermatomyositis, immune-mediated necrotizing myopathy, anti-synthetase syndrome, inclusion body myositis, overlap myositis (with another connective tissue disease) [20]. The differential diagnosis is based on clinical, immunological and histological features. The heterogeneous clinical phenotype and the challenges identifying conclusive pathogenetic mechanisms lead to proposal of three candidate ERNs (Table 5): ReCONNET (European Reference Network on Connective Tissue and Musculoskeletal Diseases), RITA (European Reference Network on Rare Immunodeficiency, Autoinflammatory and Autoimmune Diseases) and EURO-NMD (European Reference Network on Rare Neuromuscular Diseases).

**Table 5:** Polymyositis example

National RD coding			National (ISS) proposal for RD Attribution TO ERN's				Data from European file (specificcriteria_en.xls)				
Exemption code	RD Name	Group	ERN 1st national candidate	ERN 2nd national candidate	ERN 3rd national candidate	ORPHA codes national proposal	Identified ERN	Main thematic group	Sub-thematic areas		Rare or complex disease(s), condition(s) or highly specialised intervention(s)
RM0020	Polymyositis	Osteomuscular system and connective tissue disorders	ReCONNET	RITA	NMD	732	Reconnet				Polymyositis, Dermatomyositis, Anti-synthetase syndrome

*RD* rare disease, *ISS* Istituto Superiore di Sanita (Italian National Institute of Health), *ReCONNET* European reference network on connective tissue and musculoskeletal diseases, *RITA* rare immunodeficiency, autoinflammatory and autoimmune diseases network, *NMD* European reference network for rare neuromuscular diseases

Tables 6 and 7, show how Rheumatology, Immunology, Internal Medicine, Neurology and Pneumology Hospital Units register Polymyositis cases on RTMR, while not every Unit is able to certificate or treat the RD. BEST CASES, BEST CERT and BEST TP labels can all be assigned to a Rheumatology Hospital Unit. Therefore, ERN ReCONNET is chosen as most suitable ERN.

**Table 6:**
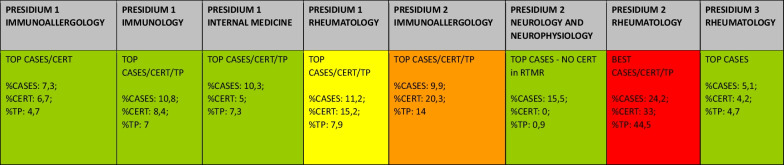
Polymyositis pivot table

The red field highlights the Presidium with the highest number of cases, certifications and treatment plans. The orange and yellow fields denote the second and third most appropriate Presidium for Polymyositis, considering number of cases, certifications and treatment plans but also the clinical, immunological and histological features of the disease

*TP* treatment plans, *CERT* certifications

**Table 7:** Polymyositis: data from Presidia in Tuscany

Single presidium RTMR data								All presidia RTMR data			Single presidium%		
Presidium (Healthcare Company-Department/Un It)		Tot CASES from 2000 (Fd or CONT)	Residents	Tot CASES from 2014 (FD or CONT)	Residents (%)	Tot CERT from 27/5/17	Tot Tp from 27/5/17	Tot CASES	Tot CERT	Tot TP	% Cases	% CERT	% Tp
Presidium 1	Immunoallergology	28	24	17	88	8	15	231	118	314	7.3	6.7	4.7
Presidium 1	Immunology	29	11	25	96	10	22	231	113	314	10.8	8.4	7.0
Presidium 1	Internal medicine	33	24	24	88	6	23	231	118	314	10.3	5.0	7.3
Presidium 1	Neurology 1	3	3	2	100	1	2	231	118	314	0.8	0.8	0.6
Presidium 1	Neurology 2	1	1	1	100	0	0	231	118	314	0.4	0.0	0.0
Presidium 1	Pneumology	4	3	4	75	0	0	231	118	314	1.7	0.0	0.0
Presidium 1	Rheumatology	31	28	26	96	18	25	231	118	314	11.2	15.2	7.9
Presidium 2	Immunoallergology	32	26	23	83	24	44	231	118	314	9.9	20.3	14.0
Presidium 2	Neurology and neurophysiopatology	57	47	36	83	0	3	231	118	314	15.5	0.0	0.9
Presidium 2	Neurology (university)	4	4	2	100	2	0	231	118	314	0.8	1.6	0.0
Presidium 2	Rheumatology	122	93	56	77	39	140	231	118	314	24.2	33.0	44.5
Presidium 3	Neurology and neurometabolic diseases	6	3	3	33	2	0	231	118	314	1.2	1.6	0.0
Presidium 3	Neurology and neurophysiopatology	6	5	5	80	0	0	231	118	314	2.1	0.0	0.0
Presidium 3	Rheumatology	19	10	12	58	5	15	231	118	314	5.1	4.2	4.7

In the table only most relevant Presidia for Kallman syndrome are shown. Inactive Presidia at the moment of data analysis have been omitted

*FD* first diagnosis, *CONT* follow-up visit, *CERT* certifications, *TP* treatment

## Results

**Fig. 1:**
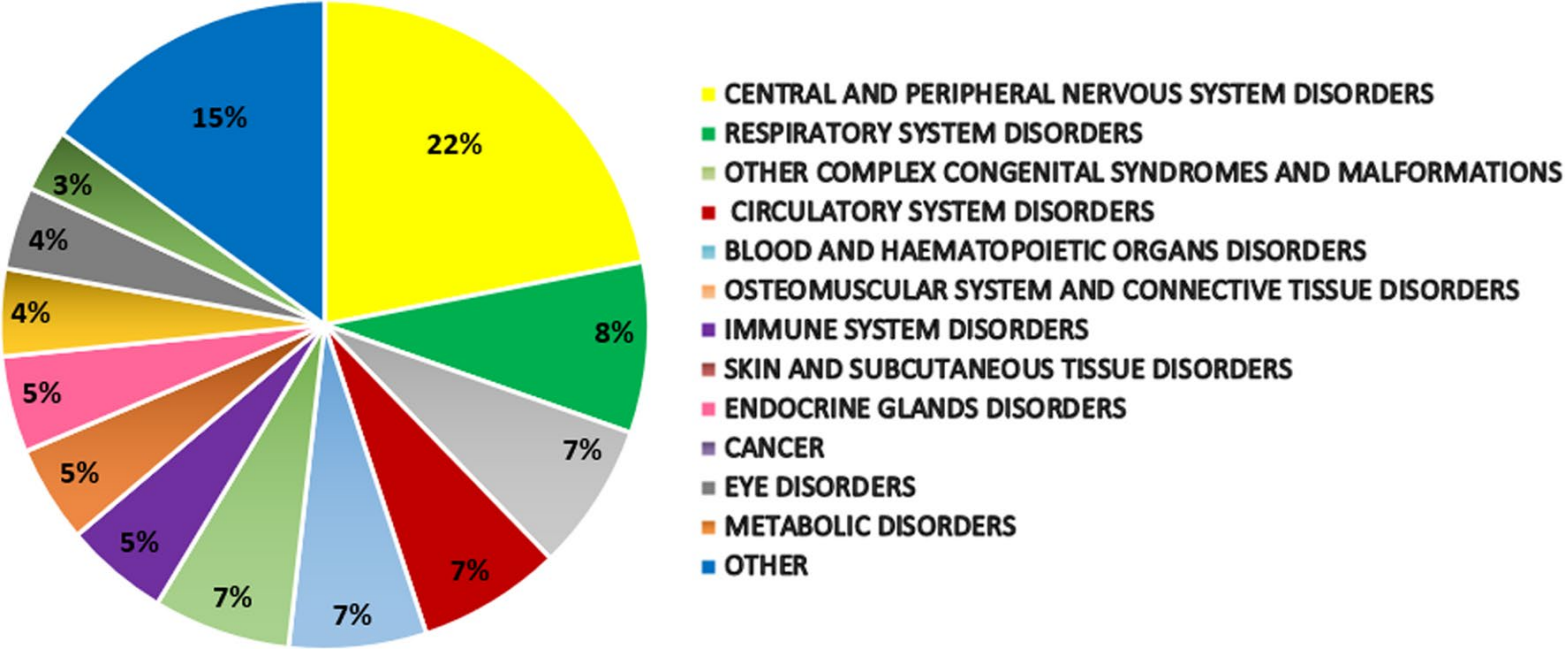
RDs according to disease group

Data analysis was performed on 60,367 cases registered in RTMR as a whole. As described in the previous section, we then chose to consider cases from 2014 to 2020, regarding 32 disease groups and subgroups, for a total of 628 RDs. Groups or subgroups of RDs with more than 1000 registered cases are shown in Fig. 1, disorders of the central and peripheral nervous system being the most prevalent group. Two-hundred and fifteen active presidia have been evaluated, as previously described.

### Regional network organisation

**Fig. 2:**
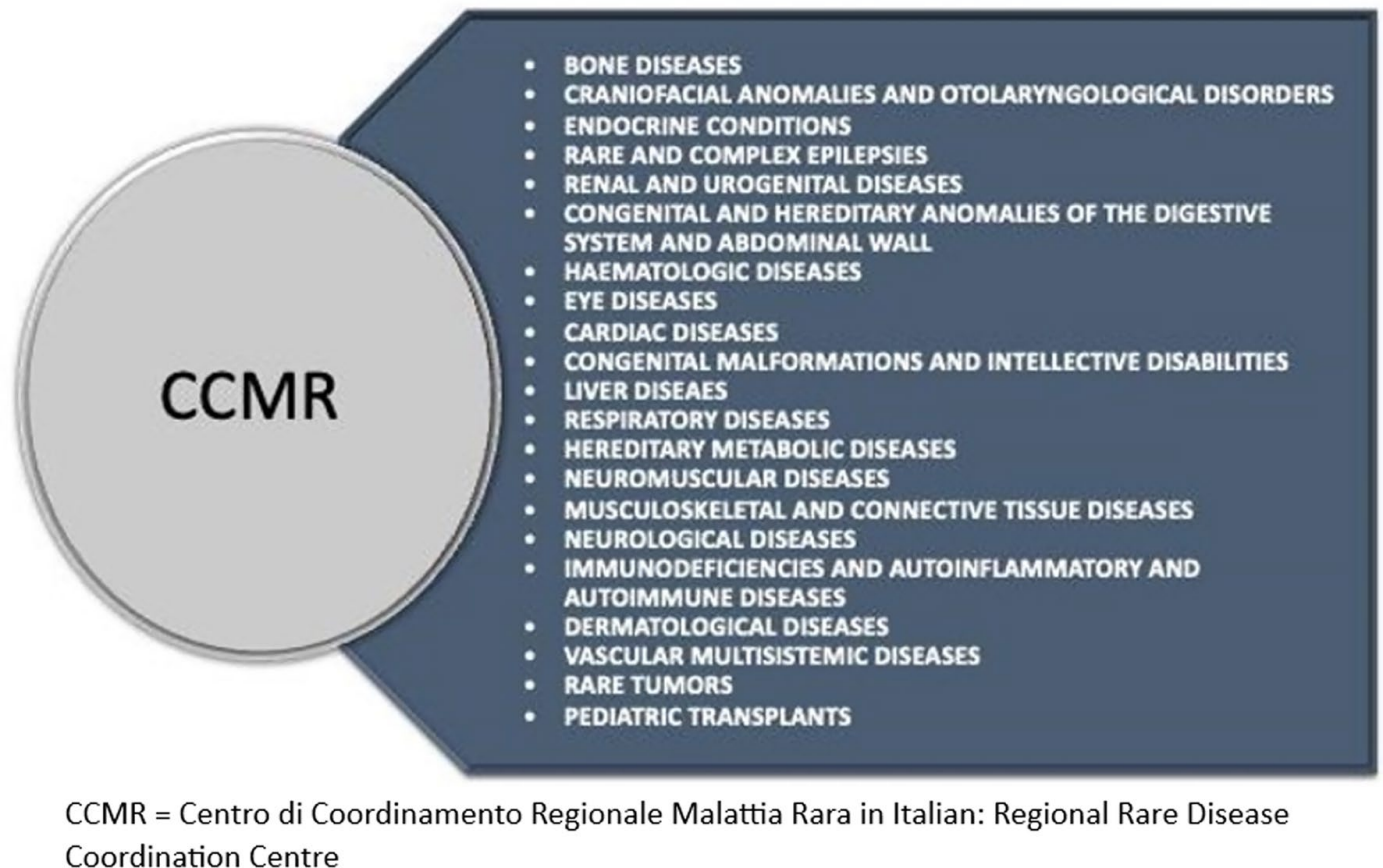
List of regional rare diseases coordination centres

After the assignment of each RD to the most suitable ERN, Regional RD Coordination Centres (CCMR, Centro di Coordinamento Regionale Malattia Rara in Italian) have been established (Fig. 2).

**Fig. 3:**
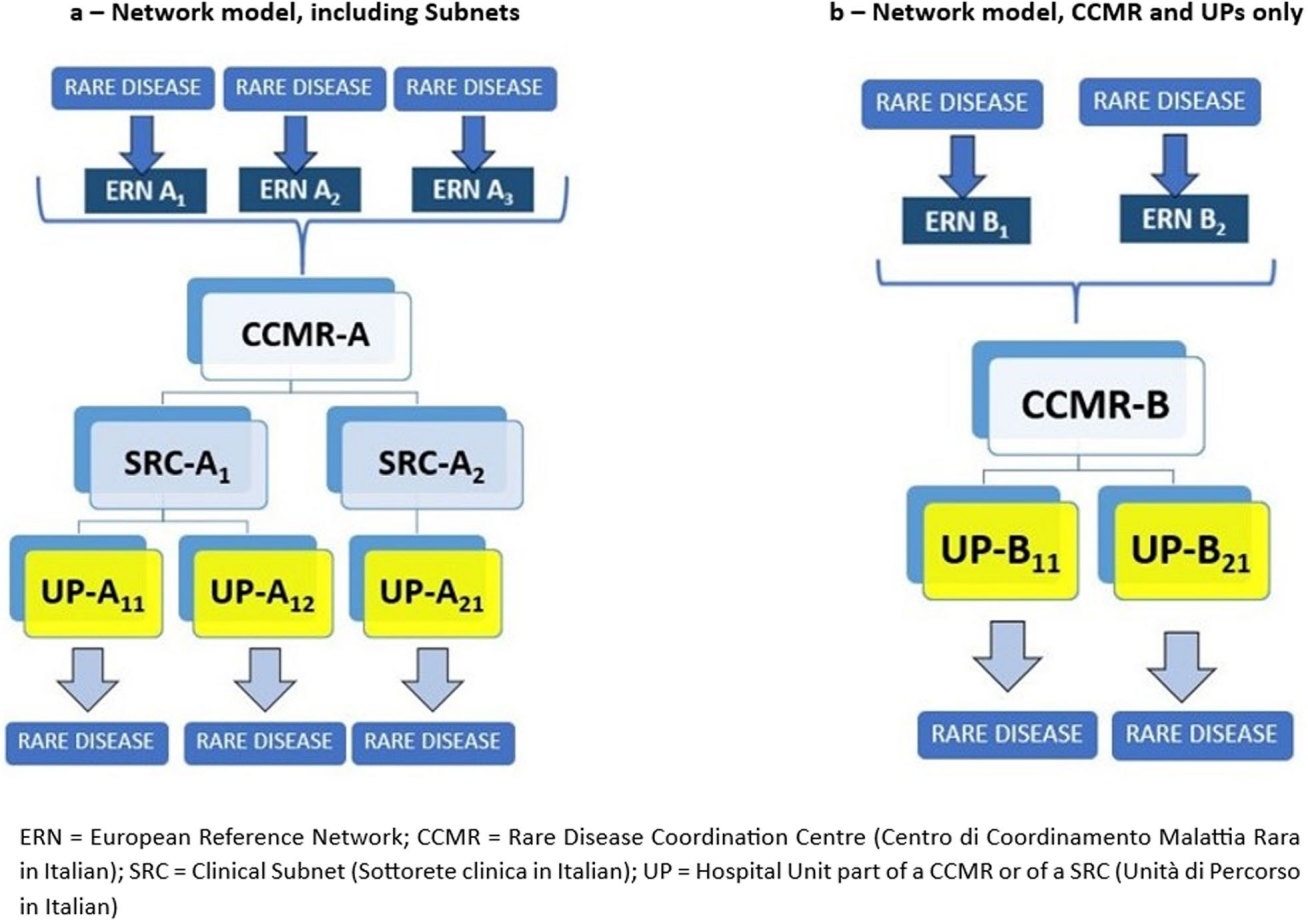
**a** Network model, including subnets. **b** Network model, CCMR and Ups only. ERN = European Reference Network; CCMR = rate disease coordination centre (Centro di Coordinamento Malattia Rara in Italian); SRC = clinical subnet (Sottorete clinica in Italian); UP = Hosptial unit part of a CCMR or of a SRC (Unità di Percorso in Italian)

A CCMR aggregates and coordinates Hospital Units (UP—Unità di Percorso in Italian) which diagnose and treat one or a group of related RDs, from a clinical and/or etiopathogenic perspective. Coordination Centres for a wide group of RDs encompass one or more Clinical Subnets (SRC, Sottorete clinica in Italian), to better manage patient’s care pathway. In Fig. 3a, b the new organisation models for the Rare Diseases Regional Network in Tuscany is graphically summarised.

### Rare diseases regional network webpage

The section “RTMR—Statistical Data” on the webpage https://malattierare.toscana.it leads to updated statistics regarding RDs in Tuscany. The data include the number of total cases entered in the Regional Registry, Registered Diseases, Active Presidia and Paediatric Cases.

Data can be filtered according to different criteria, as shown in Table 8. By clicking on the item, records are displayed both as list and in graphical representation.

**Table 8:** Freely available data on the website https://malattierare.toscana.it/

Records included in the Rare Disease Registry of Tuscany (RTMR)
Total cases by disease group
Total cases by disease
Total cases by gender
Total cases by age group
Total cases by paediatric age group
Total cases by patient's area of residence
Total cases by local health district
Total cases by Institution
Total cases by Presidium

The search can be also run by selecting a specific rare disease, the area of patient’s residency or the time span of the diagnosis (from year X to year Y).

The website features the section “Patient’s pathway” (Homepage > Patient’s pathway), where the complete list of Rare Diseases Presidia can be accessed. For each presidium, the disease-related outpatient and inpatient clinics are displayed. The Presidia Network list is a clickable item which leads to a detailed information page about the selected Presidium. Specifically, building address, list of performed medical activities, staff members contacts, laboratory procedures and website link are available.

Roles assigned to the Presidium and list of RDs records registered in RTMR are also displayed. The latter may be further expanded by clicking on the disease of interest.

For instance, selecting the first Presidium of the list (Homepage > Patient’s pathway > Presidia Network list), which is “AMYLOIDOSIS MEDICAL CLINIC” the registered cases and assigned roles are displayed. Clicking on the first disease of the list, which is “ALPORT SYNDROME”, disease’s details are available, as shown in Table 9.

**Table 9:** Alport syndrome information page

Name	Alport syndrome
Exemption code	RN1360
Group	Diseases of genito-urinary system
Definition	Alport syndrome (SA) is a hereditary disease characterized by structural defect of type IV collagen leading to hematuria and structural alterations of the glomerular basement membrane. AS is often associated with hearing and vision Impairment caused by anomalies in cochlea and crystalline
CCMR	CCMR—Renal and urogenital diseases Specialised reference institution: Name of institution—Nephrology
HEAD of the centre	Name of responsible medical specialist
Clinical sub-network	Renal pediatric diseases Specialised reference institution: Name of institution—Nephrology
Head of clinical sub-network	Name of responsible medical specialist

*CCMR* Centro di Coordinamento Regionale Malattia Rara in Italian: Regional Rare Disease Coordination Centre

Likewise, the path *Homepage* > *Patient’s pathway* leads to the complete list of the 21 Regional Coordination Centres (CCMR). For each CCMR the list of involved RDs is displayed, with respective information pages (Table 10). For example, in the case of Cardiac Rare Diseases CCMR, two clinical sub-networks are shown, Arrhythmogenic cardiopathies and Congenital Cardiopathies, which can be further expanded (Table 11).

**Table 10:** Rare cardiac diseases regional coordination centre

CCMR	CCMR cardiac diseases
Head of the centre	Name of responsible medical specialist (Specialised Reference Institution)
Clinical sub-network	Click on the clinical sub-network to display related diseases: Arrhythmogenic cardiopathies (Head: Name of responsible medical specialist), Congenital cardiopathies (Head: Name of responsible medical specialist)

*CCMR* Rare Disease Coordination Centre (Centro di Coordinamento Malattia Rara in Italian)

**Table 11:** Congenital cardiopathies clinical sub-network

Clinical subnetwork	Congenital cardiopathies (part of CCMR_CARDIAC diseases)
Head of clinical subnetwork	Name of responsible medical specialist (Specialised Reference Institution)
Sub-network's related diseases	RNG141-Criss-cross heart RNG141-Ebstein, anomaly of RNG141-Hypoplasic left heart syndrome RNG141-Severe and invalidant congenital malformation syndromes of the heart and large vessels

*CCMR* Rare Disease Coordination Centre (Centro di Coordinamento Malattia Rara in Italian)

**Fig. 4:**
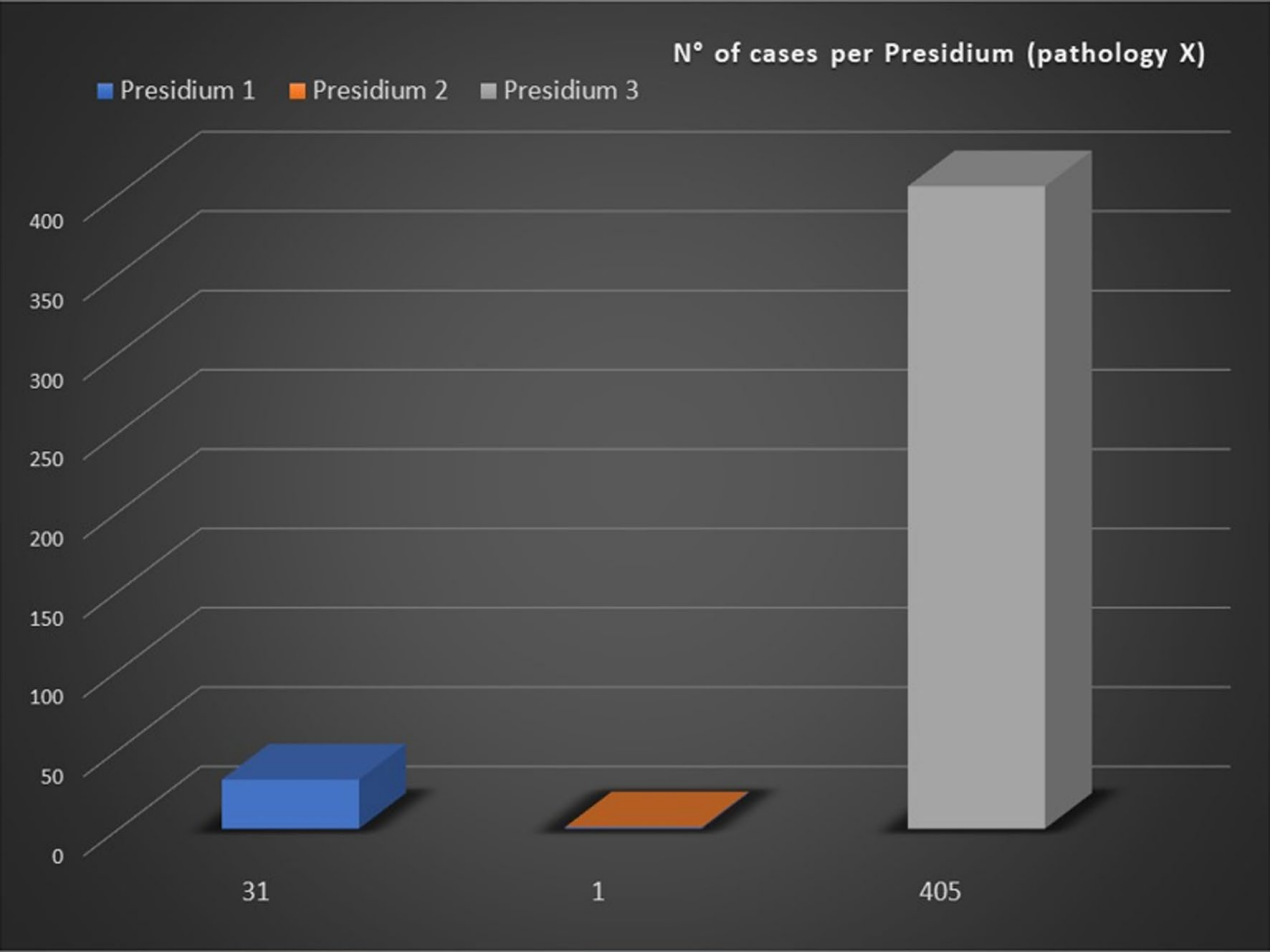
Example of graphic representation of total Rare Disease’e cases registered by Presidia

**Fig. 5:**
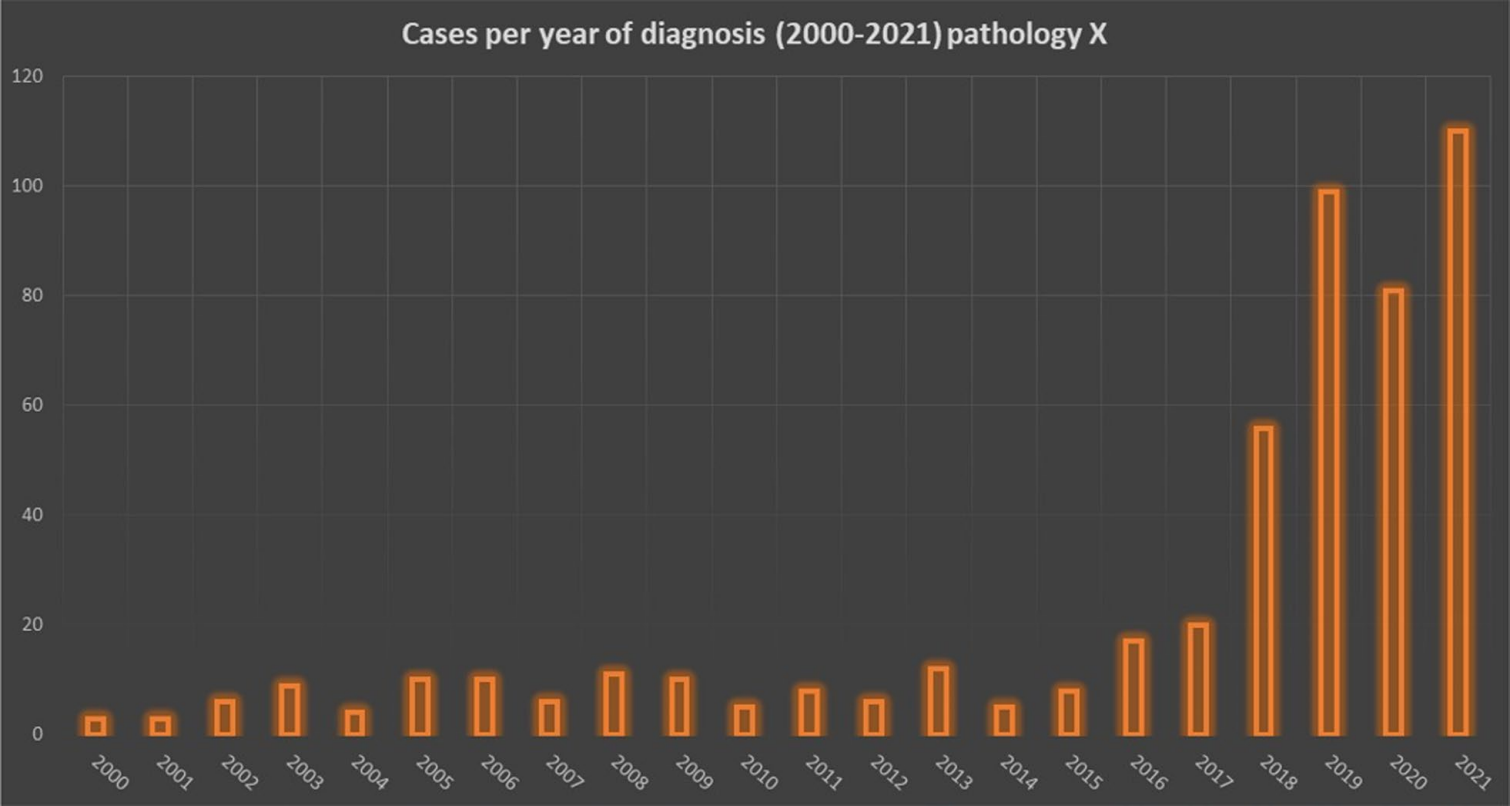
Example of graphic representation of cases registered by Presidia by year

Statistics are available for each Clinical Subnetwork’s disease by clicking on it and selecting the “Statistical Data” item on the top left of the webpage. For each RD, the number of total cases registered in Tuscany Institutions is reported and further information are displayed both as basic list and in graphical form, as shown in Figs. 4 and 5 (about an unspecified disease named “X”).

### Rare diseases regional network approval

The new Regional Network of Rare Diseases was approved by Regional Decree n. 4234/2021 and further updates [21] and it was published and made freely accessible on the previously mentioned website in June 2021. The synthesis of this work, relevant data about Presidia (Pivot tables and labels) and the subsequent Network structure was also disseminated among Health Institutions in Tuscany. Regional Decrees are regularly updated according to the network evolution.

## Discussion

The new Tuscany Rare Diseases Regional Network was conceived and built as a system of interconnections among medical facilities ensuring highly specialised medical care in the field of RDs. The selection of Hospital Units coordinated by Regional Coordination Centres and possible Clinical Subnetworks reflect the concept of a Network based on homogenous groups of RDs. This model guarantees patients’ referral to high competence Institutions and the creation of specific diagnostic and treatment pathways, reducing misdiagnosis, delayed diagnosis and improper treatment, which are significant issue in the field of RDs.

The establishment of ERNs represented an extremely valuable model to plan the Regional Network, but it was a tough challenge to coherently translate ERNs’ structural organisation into the regional system.

In fact, the assignment of each RD to the appropriate Presidium had to be made considering not only the number of registered cases, certifications and treatment plans but also the competence in multidisciplinary management of the patient, from diagnosis to treatment. Moreover, the weight of different criteria often depends on the considered RD. For example, patients affected by multisystemic diseases need Presidia that can assure effective collaboration among professionals. That is an essential requirement also for very rare diseases which need specialised care from early diagnosis to innovative therapies.

Therefore, the chosen Coordination Centre has to identify and supervise the most appropriate pathway for the diagnosis and follow-up of patients with RDs, ensuring their referral to Presidia that can provide effective diagnostic tools, a global medical follow-up and latest available treatments.

One of the strengths of the Regional Network is its dynamism, ensured by the active role of medical experts in updating the RTMR. Transparency is another peculiarity of Tuscany Network: data are shared through a freely accessible website, allowing patients to easily retrieve information about their disease, regional diagnostic and treatment pathways and contact details to start or carry on their medical care. This is also useful to medical staff and clinical researchers to refer their patient affected by RDs to the appropriate Centre or to ask for consultation. Patient’s Associations are also strongly involved in all the steps leading to the building of Regional Network.

### Limitations

The Rare Disease Regional Network in Tuscany is based on the data from the RTMR registry, a tool which is daily updated by health professionals. Therefore, the number of cases for each RD is highly dependent on the accurate data entry and it could be underestimated. Moreover, data regarding certifications and treatment plans are only available since 2017. Research on rare diseases is rapidly evolving, hence this kind of reorganisation process should be carried out frequently to ensure that the Network is updated for each RD, including recently identified disorders and Presidia which can provide the latest therapeutic options.

## Conclusions

The current organisation of Tuscany Rare Diseases Regional Network is the result of more than twenty years of work, since the approval of Italian Ministerial Decree 279/2001 in 2001 [14], thanks to the regional policies oriented to answer RDs patients’ needs. Healthcare system and clinical researchers need specific, dynamic and innovative pathways to improve diagnosis, follow-up and treatment of RDs.

From this perspective, the 2020 remapping has led to a solid Network based on European Reference Networks, to facilitate collaboration on a Regional, National and International level.

This system is dynamic and subject to implementation, following not only scientific research but also the development of Presidia’s expertise. The monitoring and update of the Network is ensured by all the actors who play a role in Rare Diseases: Regional Coordination Group for Rare Diseases, healthcare specialists and professional, the Forum for RDs patients’ Associations, with the essential support of the Tuscany Rare Diseases Registry.

## Data Availability

The complete datasets generated and analysed during the current study are not publicly available due to the presence of sensitive data regarding regional healthcare institutions but are available from the corresponding author on reasonable request (requests can also be sent through RTMR contact form: https://malattierare.toscana.it/informazioni/contatti/). Source of data is publicly available and accessible on https://malattierare.toscana.it/dati-statistici/. Detailed data about Presidia’s RDs cases are stored in the RTMR restricted access area https://registripatologia.ftgm.it/rtmr/.
